# *Mcam* Silencing With RNA Interference Using Magnetofection has Antitumor Effect in Murine Melanoma

**DOI:** 10.1038/mtna.2014.56

**Published:** 2014-10-28

**Authors:** Lara Prosen, Bostjan Markelc, Tanja Dolinsek, Branka Music, Maja Cemazar, Gregor Sersa

**Affiliations:** 1 Kolektor Group, Nanotesla Institute Ljubljana, Ljubljana, Slovenia; 2 Department of Experimental Oncology, Institute of Oncology Ljubljana, Ljubljana, Slovenia; 3 Department of Natural and Medical Subjects, University of Primorska, Faculty of Health Sciences, Izola, Slovenia

## Abstract

The melanoma cell adhesion molecule (MCAM) is involved in melanoma development and its progression, including invasiveness, metastatic potential and angiogenesis. Therefore, MCAM represents a potential target for gene therapy of melanoma, whose expression could be hindered with posttranscriptional specific gene silencing with RNA interference technology. In this study, we constructed a plasmid DNA encoding short hairpin RNA against MCAM (pMCAM) to explore the antitumor and antiangiogenic effects. The experiments were performed *in vitro* on murine melanoma and endothelial cells, as well as *in vivo* on melanoma tumors in mice. The antiproliferative, antimigratory, antiangiogenic and antitumor effects were examined after gene therapy with pMCAM. Gene delivery was performed by magnetofection, and its efficacy compared to gene electrotransfer. Gene therapy with pMCAM has proved to be an effective approach in reducing the proliferation and migration of melanoma cells, as well as having antiangiogenic effect in endothelial cells and antitumor effect on melanoma tumors. Magnetofection as a developing nonviral gene delivery system was effective in the transfection of melanoma cells and tumors with pMCAM, but less efficient than gene electrotransfer in *in vivo* tumor gene therapy due to the lack of antiangiogenic effect after silencing *Mcam* by magnetofection.

## Introduction

A contemporary approach in cancer gene therapy is to target specific molecules or signaling pathways that are predominantly expressed in tumor cells, and is therefore expected to have a good therapeutic index.^1^ The introduction of genetic material into target cells, with an effective and safe delivery system, still remains a challenge in the development of new gene therapy strategies. Several viral and nonviral transfection approaches are being investigated. The use of viral vectors provides specificity, efficiency, long term expression, stability and integrity, however due to safety issues, the development of new nonviral delivery systems is gaining in value.^2^

Among the nonviral delivery systems that hold promise, gene electrotransfer is becoming well acknowledged, providing the physical method that can safely and effectively transfect cells *in vitro* and tumors *in vivo* by applying high-voltage electric pulses of defined magnitude and length.^3^ Several studies have demonstrated effective gene electrotransfer of reporter and therapeutic genes to tumors, skin, muscle, and other tissues.^4,5,6,7^ Nevertheless, the method still needs refinement, with several aspects being investigated, either the reduction of ROS production by gene electrotransfer or interfering with the endocytic pathway that is, at least partially, also involved in plasmid DNA uptake with gene electrotransfer.^8,9^ Magnetofection is another nonviral gene delivery method, where functionalized superparamagnetic iron oxide nanoparticles (SPIONs) coupled with nucleic acids are used and guided by an external magnetic field to the targeted cells, in order to facilitate the introduction of nucleic acids into the cells.^10^ We and others have already demonstrated that magnetofection is an efficient nonviral transfection method *in vitro* and *in vivo* for reporter and therapeutic genes.^11,12,13^ Besides plasmid DNA, also small interfering RNA (siRNA), short hairpin RNA (shRNA) and antisense oligonucleotides can be used for the magnetofection of cells.^14,15,16^ The advantage of magnetofection is that it is a noninvasive method, as surface magnets are used to deliver the therapeutic plasmid DNA into the targeted cells, and, compared to gene electrotransfer, is also a painless method, that may have better compliance for patients when translated into clinics. However, gene electrotransfer was demonstrated as a more effective method in the transfection of tumors with reporter plasmid DNA for enhanced green fluorescent protein (pEGFP) compared to magnetofection.^11^

The melanoma cell adhesion molecule (MCAM) is a multi-functional transmembrane glycoprotein that has an important role in the development of melanoma as well as its progression, including invasiveness, metastatic potential and vascular angiogenesis. Its overexpression was confirmed in a variety of tumors, like melanoma, breast, prostate, ovarian and lung cancer, therefore it represents a promising target for the treatment of tumors.^17^ Various approaches for targeting MCAM have already been investigated; antibodies against MCAM, immunization with MCAM, silencing of *Mcam* expression with siRNA and shRNA molecules.^18,19,20,21,22,23,24,25,26,27^ The use of antibodies against MCAM has been effective *in vitro* in the reduction of proliferation, migration and tube formation in human endothelial cells,^18,19^ and invasion in melanoma and osteosarcoma cells.^19,20^ The therapy of melanoma, osteosarcoma, hepatocarcinoma, leiomyosarcoma and pancreatic tumors in mice *in vivo* with intraperitoneal injection of antibodies against MCAM reduced tumor growth and the formation of metastases of melanoma and osteosarcoma.^18,19,20^ Another approach, silencing of *Mcam* expression with small noncoding RNA molecules, has also demonstrated a promising effect in human melanoma, endothelial, breast cancer, ovarian cancer and adenoid cystic carcinoma cell lines *in vitro*. It reduced proliferation or survival, adhesion, migration and invasion of cells.^22,23,24,25,26^ There was only one *in vivo* study targeting MCAM mRNA by RNA interference technology (RNAi), where the treatment of pathological angiogenesis in mice with miRNA targeting MCAM mRNA significantly attenuated neovascularization.^27^ A step further in this field would definitely be the use of plasmid DNA encoding shRNA against MCAM, which allows more stable and long-lasting expression of shRNA for the maximization of therapeutic effect in comparison with siRNA delivered alone.^28^ Therefore, the aim of our study was to construct a new plasmid DNA encoding shRNA against MCAM, which was then thoroughly tested on antiproliferative, antimigratory and antiangiogenic effects in murine melanoma and endothelial cells *in vitro*. As the first, we extended the investigations on antitumor effect of newly constructed plasmid DNA after magnetofection and gene electrotransfer in melanoma tumors in mice. Furthermore, we compared the effectiveness of magnetofection as a developing transfection method to gene electrotransfer as an established method.

## Results

### Selection of the most effective siRNA molecule against MCAM and the construction of the plasmid DNA encoding shRNA against MCAM

Three selected siRNA molecules (siRNA 801, siRNA 802, and siRNA 552) were transfected into B16F1, B16F10, and 2H-11 cells with lipofection in order to determine their effect on MCAM mRNA and protein levels, as well as their influence on the biological properties of cells *in vitro*.

The qRT-PCR analysis showed a very similar expression of MCAM mRNA in untreated B16F1, B16F10, and 2H-11 cells (Ct values for MCAM mRNA were 23.40, 23.99, and 22.88, and for internal reference 18S rRNA 8.85, 8.61, and 8.14, respectively).

**Figure 1:**
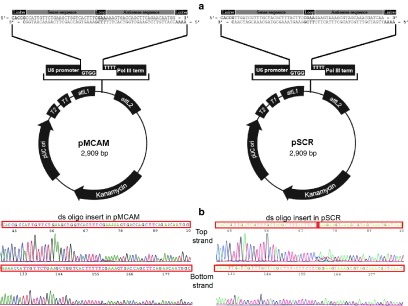
The constructed therapeutic plasmid pMCAM and control plasmid pSCR. (**a**) The map and the features of constructed plasmid DNAs (U6 cassette, which contains the elements required for controlled expression of shRNA in the cells; cloning site; ds oligo insert encoding shRNA; two recombination sites, *att*L1 and *att*L2; kanamycin resistance gene for selection in *E. coli*; pUC origin for high-copy maintenance of plasmid DNA in *E. coli*) of the pENTR/U6 vector with double-stranded oligonucleotides (ds oligo) encoding shRNA against MCAM (left) or with scrambled nucleotide sequence (right). (**b**) Sequencing of the top and bottom strands of a ds oligo insert in pMCAM (left) and pSCR (right) to confirm the presence and correct orientation of the ds oligo insert.

Among all tested siRNA molecules, siRNA 801 was the most effective in reducing MCAM mRNA (**Supplementary Figure S1**) and protein levels (**Supplementary Figure S2**), decreasing the survival of B16F1 and B16F10 cells (**Supplementary Figures S3 and S4**), reducing the migration of B16F10 cells (**Supplementary Figures S5 and S6**) and the ability of 2H-11 cells to form capillary-like structures (**Supplementary Figures S7 and S8**). Therefore, its nucleotide sequence was selected for further construction of plasmid DNA encoding shRNA against MCAM (pMCAM). All results with siRNA's are presented in **Supplementary Material**. In addition to the preparation of pMCAM, also a control plasmid DNA encoding shRNA with scrambled nucleotide sequence of siRNA 801 (pSCR) was constructed (**Figure 1a**). By sequencing of both plasmids the presence, correct orientation and correct sequence of the ds oligo inserts were confirmed (**Figure 1b**).

### Transfection efficiency of magnetofection and gene electrotransfer

**Figure 2:**
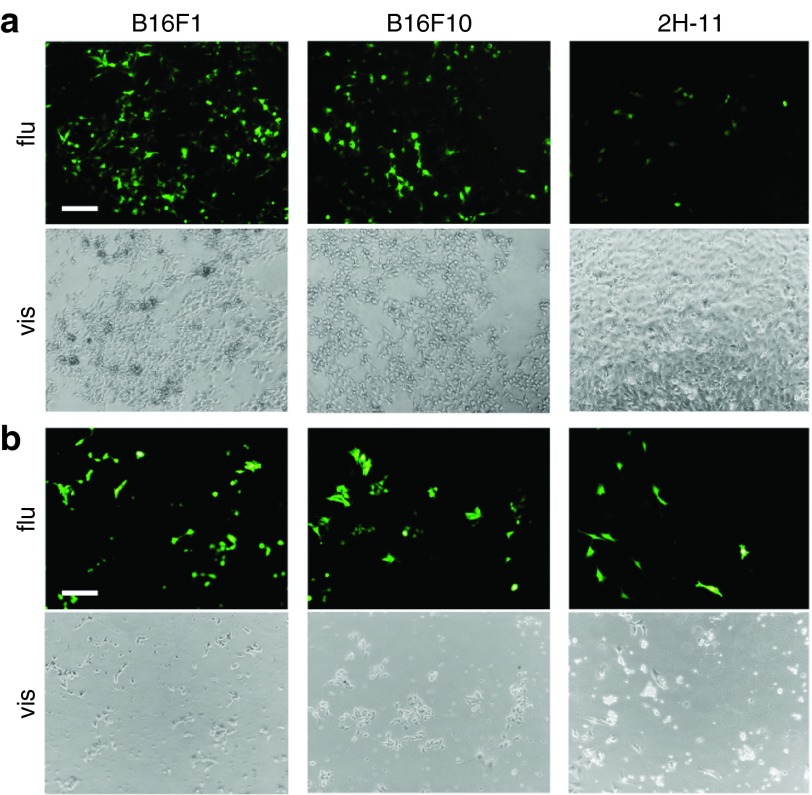
The expression of enhanced green fluorescent protein (EGFP) in the cells visualized by fluorescence microscopy. The expression of EGFP obtained 48 hours after (**a**) magnetofection and (**b**) gene electrotransfer in B16F1, B16F10, and 2H-11 cells. The first and third rows of images represent cells under fluorescent light (flu) (excitation 460–490 nm and emission filters 515 nm long pass), the second and fourth rows under visible light (vis). The images were taken under 10× magnification. Scale bar, 200 µm.

To determine the transfection efficiency of magnetofection and gene electrotransfer in B16F1, B16F10, and 2H-11 cells, the reporter plasmid pEGFP was used for transfection. The images, obtained 48 hours after transfection, demonstrated effective transfection of cells by both methods (**Figure 2**). B16F1 cells were comparably transfected by both methods, while B16F10 cells were done so slightly better by gene electrotransfer. The main difference in the effectiveness between both methods was observed in 2H-11 cells, since the amount of cells expressing EGFP was higher after gene electrotransfer than after magnetofection.

### The effect of transfection with pMCAM on the biological properties of cells *in vitro*

The constructed pMCAM was tested for its effectiveness in the reduction of MCAM mRNA and protein levels in melanoma and endothelial cells. Furthermore, the biological properties were tested, such as cell proliferation, migration of melanoma cells and the ability of endothelial cells to form capillary-like structures for a potential antiangiogenic effect. Magnetofection as a developing gene delivery system was explored and compared to gene electrotransfer as an established method.

**Figure 3:**
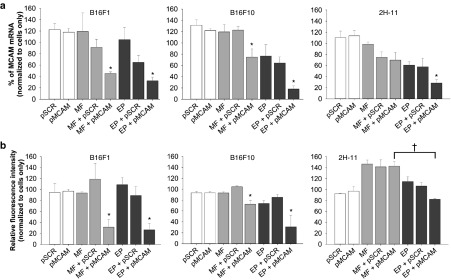
The MCAM mRNA and MCAM protein levels in B16F1, B16F10, and 2H-11 cells examined after magnetofection and gene electrotransfer with pMCAM. (**a**) The MCAM mRNA levels determined by qRT-PCR analysis and (**b**) MCAM protein levels determined by flow cytometer analysis in B16F1, B16F10, and 2H-11 cells 48 hours after magnetofection (MF) or gene electrotransfer (EP) with pMCAM. Bars represent AM ± SEM of the percentage of MCAM mRNA (*N* = 3) or the relative fluorescence intensity (*N* = 3). Asterisks indicate statistically significant differences between five control groups (untreated cells, only pSCR or pMCAM separately added to cells, MF or EP, MF + pSCR or EP + pSCR) and the group compared (MF + pMCAM or EP + pMCAM) (*P < 0.05). Crosses indicate statistically significant differences between MF + pMCAM and EP + pMCAM (^†^*P* < 0.05). All data were normalized to MCAM mRNA or the fluorescence intensity of untreated cells labeled with FITC-MCAM antibodies.

*Reduction of MCAM mRNA and subsequent MCAM protein levels.* To determine if transfection with pMCAM reduced the MCAM mRNA level, total mRNA was isolated from B16F1, B16F10, and 2H-11 cells 48 hours after magnetofection or gene electrotransfer, and a qRT-PCR analysis followed. The MCAM mRNA levels were statistically significantly decreased in B16F1 and B16F10 cells after magnetofection and gene electrotransfer; however, in 2H-11 cells, the level of MCAM mRNA was statistically significantly reduced only after gene electrotransfer (**Figure 3a**). The level of MCAM mRNA was reduced for 55% in B16F1, 25% in B16F10, and 30% in 2H-11 cells after magnetofection and for 67% in B16F1, 82% in B16F10, and 72% in 2H-11 cells after gene electrotransfer. Furthermore, immunocytofluorescence was used to determine MCAM protein levels 48 hours after transfection with flow cytometry. The expression of MCAM at the protein level was statistically significantly decreased in B16F1 and B16F10 cells after magnetofection and gene electrotransfer. The level of MCAM protein was reduced for 69% in B16F1 and 27% in B16F10 cells after magnetofection and for 74% in B16F1 and 69% in B16F10 cells after gene electrotransfer. Gene electrotransfer was effective also in reducing the MCAM protein level in 2H-11 cells for 17%, which was statistically significantly lower in comparison to MCAM protein level after magnetofection (**Figure 3b**). Transfection with pSCR had no statistically significant effect on MCAM mRNA and protein level in any of the tested cell lines.

**Figure 4:**
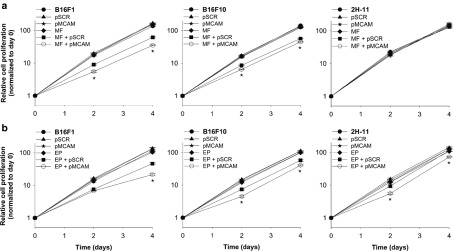
The effect of *Mcam* silencing on cell proliferation. The proliferation of B16F1, B16F10, and 2H-11 cells after (**a**) magnetofection (MF) and (**b**) gene electrotransfer (EP) with pMCAM was monitored for 4 days. All data for cell proliferation curves are expressed as AM ± SEM (*N* = 42). Asterisks indicate statistically significant differences between five control groups (untreated cells, only pSCR or pMCAM separately added to cells, MF or EP, MF + pSCR or EP + pSCR) and the group compared (MF + pMCAM or EP + pMCAM) (*P < 0.05). All data are normalized to day 0.

*Antiproliferative effect of Mcam silencing.* Cell proliferation was measured up to 4 days after *Mcam*. silencing by magnetofection or gene electrotransfer with pMCAM. The proliferation of B16F1 and B16F10 cells was significantly inhibited by magnetofection and gene electrotransfer, whereas the latter was also effective in 2H-11 cells. Magnetofection resulted in a statistically significant inhibition of cell proliferation for 77% in B16F1 and 65% in B16F10 cells at day 4 (**Figure 4a**). The inhibition of cell proliferation after magnetofection in B16F1 and B16F10 cells was comparable (without statistically significant differences) to that after gene electrotransfer, which was 79% at day 4 in B16F1 cells and 59% in B16F10 cells (**Figure 4b**). Gene electrotransfer also significantly decreased the proliferation in 2H-11 cells for 32% at day 4. Transfection with pSCR did not significantly reduce proliferation in any tested cell line.

**Figure 5:**
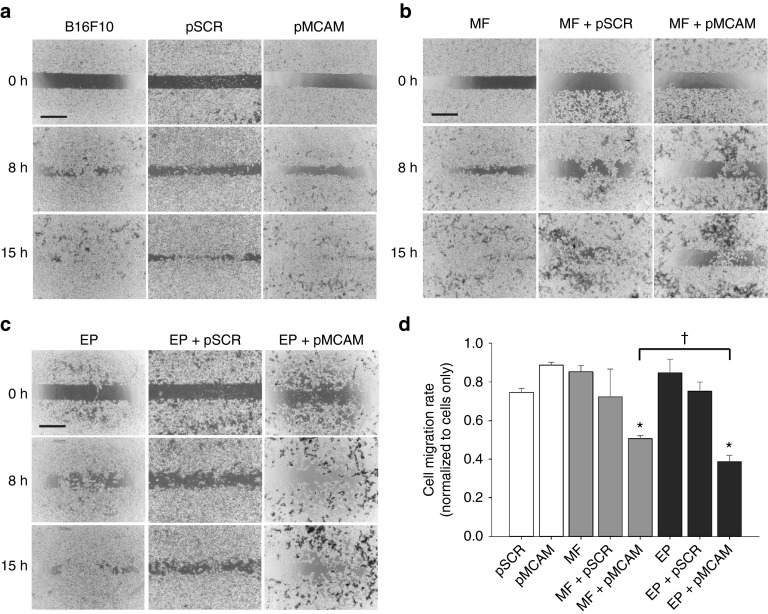
The effect of *Mcam* silencing on migration of B16F10 cells. (**a-c**) The images of a wound healing assay were taken 48 hours after magnetofection (MF) and gene electrotransfer (EP) with pMCAM under visible light, 4× magnification and at 0, 8, and 15 hours, as designated, after the removal of a silicone cell-separator. Scale bar, 500 µm. (**d**) The kinetic analysis of cell migration. Bars represent AM ± SEM of the cell migration rate (*N* = 5). Asterisks indicate statistically significant differences between five control groups (untreated cells, only pSCR or pMCAM separately added to cells, MF or EP, MF + pSCR or EP + pSCR) and the group compared (MF + pMCAM or EP + pMCAM) (*P < 0.05). Crosses indicate statistically significant differences between MF + pMCAM and EP + pMCAM (^†^*P* < 0.05). All data are normalized to the untreated cells.

*Antimigratory effect of Mcam silencing.* We examined the potential antimigratory effect of *Mcam* silencing by magnetofection and gene electrotransfer with pMCAM in B16F10 cells 48 hours after transfection. The performed wound healing assay was inappropriate for B16F1 cells, because they started to migrate barely 30 hours after the removal of silicon cell-separator, and during this time they grew in a multilayer and consequently died (data not shown). Already from the images taken at 0, 8, and 15 hours after the removal of the silicon cell-separator, a difference in the cell migration rate between experimental and control groups was noticed (**Figure 5a**–**c**). There was almost no cell-free area left in the control groups after 15 hours. However, after magnetofection and gene electrotransfer with pMCAM, the cell-free area contained fewer migratory cells. The kinetic analysis of cell migration confirmed that *Mcam* silencing by magnetofection or gene electrotransfer with pMCAM statistically significantly reduced the cell migration rate (**Figure 5d**). The migration of cells was slowed down by a factor of 2 by magnetofection and 2.5 by gene electrotransfer, with the latter being statistically significantly more effective compared to magnetofection. Transfection with pSCR had no effect on the migration of B16F10 cells.

**Figure 6:**
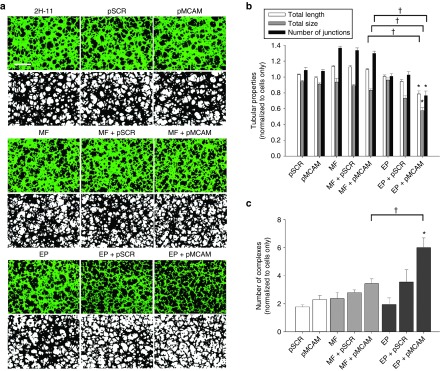
The effect of *Mcam* silencing on the ability of 2H-11 cells to form capillary-like structures. (**a**) The images of the tube formation assay were taken 48 hours after magnetofection (MF) and gene electrotransfer (EP) with pMCAM. The images in the first, third and fifth rows were taken under fluorescent light (excitation 460–490 nm and emission filters 515 nm long pass) and 4× magnification. The second, fourth, and sixth rows represent the binary images, which were made from raw images for the quantification of the total length and size of tubular complexes, the total number of junctions and the number of complexes. Scale bar, 500 µm. (**b, c**) The quantitative data of the tubular properties (length, size, number of junctions) and the number of complexes obtained after the analysis of binary images. Bars represent AM ± SEM of the tubular properties (*N* = 8). Asterisks indicate statistically significant differences between five control groups (untreated cells, only pSCR or pMCAM separately added to cells, MF or EP, MF + pSCR or EP + pSCR) and the group compared (MF + pMCAM or EP + pMCAM) (*P < 0.05). Crosses indicate statistically significant differences between MF + pMCAM and EP + pMCAM (^†^*P* < 0.05). All data are normalized to the untreated cells.

*Antiangiogenic effect of Mcam silencing.* Since MCAM is known to be involved in tumor angiogenesis, we have investigated the potential antiangiogenic effect of *Mcam* silencing with a tube formation assay, performed 48 hours after magnetofection and gene electrotransfer of 2H-11 cells with pMCAM. Untreated 2H-11 cells formed capillary-like structures (tubular complex) 2.5 hours after seeding them on Matrigel. Already from the images taken under fluorescent light after the addition of Calcein AM, the most notable effect on the ability of forming capillary-like structures can be observed after gene electrotransfer with pMCAM (**Figure 6a**). The formed capillary-like structures were smaller, with an unusual shape and with impaired cell-to-cell contacts. There were also numerous separate cells observed, excluded from formed capillary-like structures. Magnetofection with pMCAM had no apparent effect on the formation of capillary-like structures in 2H-11 cells. Furthermore, the analysis of images demonstrated that after gene electrotransfer with pMCAM the total length, size, and number of junctions of tubular complexes were statistically significantly reduced in comparison to untreated cells for 21, 23, and 42%, respectively (**Figure 6b**). Also the number of complexes increased significantly sixfold (**Figure 6c**). Magnetofection with pMCAM had no significant effect on the measured properties of tubular complexes. Transfection with pSCR had no significant effect on the ability of 2H-11 cells to form capillary-like structures.

### Antitumor effect of *Mcam* silencing in B16F10 murine melanoma tumors *in vivo*

**Figure 7:**
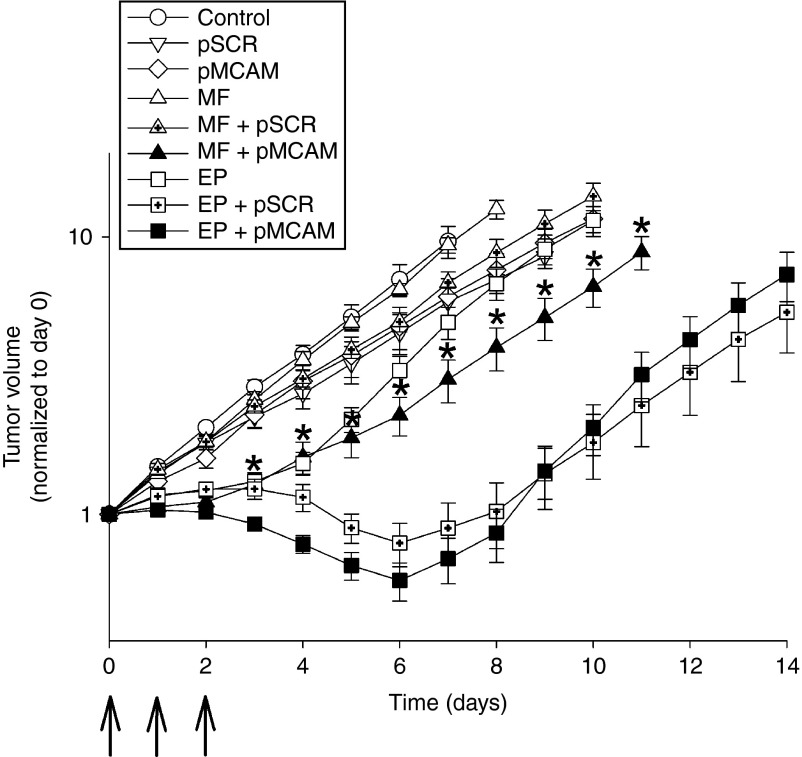
Magnetofection (MF) and gene electrotransfer (EP) of B16F10 murine melanoma tumors with pMCAM. The growth of B16F10 tumors in C57Bl/6 mice after three consecutive treatments. Arrows indicate the day of the treatments. Only mice without a complete response were included in the calculations for the tumor growth curve. Error bars represent AM ± SEM of the tumor volume (*N* = 6–12). Asterisks indicate statistically significant differences between MF + pMCAM and EP + pMCAM (*P < 0.05). All data are normalized to day 0.

In order to test the feasibility and efficacy of magnetofection in comparison to gene electrotransfer *in vivo*, gene therapy of B16F10 tumors with pMCAM was performed. Three consecutive treatments with magnetofection of tumors resulted in the stabilization of tumor growth during the time of treatment. The antitumor effect was statistically significant, producing 4.2 ± 1.0 days tumor growth delay (**Figure 7** and **Table 1**). Magnetofection of tumors with control plasmid pSCR had no antitumor effect. Gene electrotransfer was more effective than magnetofection; the tumors regressed and started to regrow at day 7 after the beginning of the therapy. Tumor growth delay was 8.0 ± 0.5 days and 17% of the treated tumors were cured (complete response). Gene electrotransfer with control plasmid pSCR also had some antitumor effect, which was comparable to the therapeutic plasmid pMCAM but with less effect during the time course of the treatment and an additional 4 days after the treatment (**Table 1** and **Figure 7**), as was already observed in other studies.^4^

## Discussion

The results of this study demonstrate that MCAM is a valuable therapeutic target in melanoma tumors, the level of which can be effectively reduced at mRNA and protein levels by a plasmid DNA encoding shRNA against MCAM. Our *in vitro* and *in vivo* data on silencing of *Mcam* showed antiproliferative and antimigratory effects in murine melanoma cells and antiangiogenic effects in murine endothelial cells, and moreover antitumor effect in the murine melanoma tumor model. Magnetofection as a developing gene delivery system has proved to be effective in transfection of melanoma cells and tumors with therapeutic plasmid DNA, pMCAM, but less efficient than gene electrotransfer in gene therapy of tumors *in vivo*.

Although silencing with siRNA or vector-based shRNA achieves similar functional outcomes, their applicability differs. Because siRNA molecules are extremely unstable and susceptible to enzymatic degradation, providing only transient gene silencing, which disappears within several days after transfection, the utilization of vector-based shRNA, which allows longer and more stable gene silencing, is recommended for *in vitro* and especially *in vivo* assays.^28,29^ However, the benefit of construction of vector-based shRNA over siRNA is not clear in all cases,^30^ especially in the case of therapeutic genes. Furthermore, the refinement of nonviral methods in order to provide better transfection efficiency in tumors, could demonstrate the advantages of vector-based shRNA over siRNA. However, we constructed a plasmid DNA encoding shRNA against MCAM (pMCAM), based on the nucleotide sequence of the most effective siRNA molecule against MCAM, and used it in *in vitro* and *in vivo* assays.

For the reduction of targeted mRNA level, besides the successful construction of therapeutic plasmid DNA an efficient gene delivery system is also needed. In this study, magnetofection as a developing nonviral transfection method was investigated, and gene electrotransfer as a reference method used.^31,32^

In our previous study, diverse magnetofection efficiency among different cells lines was demonstrated.^11^ Therefore, in this study firstly the differences in magnetofection efficiency among B16F1, B16F10, and 2H-11 cells were explored with reporter plasmid pEGFP. Magnetofection proved to be effective in transfection of B16F1 and B16F10 cells but much less in 2H-11 cells. Additionally, gene electrotransfer with pEGFP again demonstrated high transfection efficiency in all three tested cell lines.^33^

Further on, magnetofection proved to be efficient and comparable to gene electrotransfer in the delivery of pMCAM into B16F1 and B16F10 cells *in vitro*, since the mRNA and protein levels for MCAM were similarly reduced. The obtained overall silencing efficiency with pMCAM was similar to another study, where the MCAM mRNA levels were reduced for 90–80% in breast cancer cells after transfection of vector encoding shRNA against MCAM, which targeted a different nucleotide sequence of MCAM mRNA as shRNA in pMCAM, with reagent Fugen©, a mixture of lipids and other components.^25^ However, the difference in reduction of MCAM mRNA and protein levels between magnetofection and gene electrotransfer occurred in 2H-11 cells. Gene electrotransfer significantly silenced the *Mcam* gene also in 2H-11 cells, whereas magnetofection did not. Poor silencing efficiency after magnetofection could be explained by poor magnetofection efficiency in 2H-11 cells, which was already demonstrated using reporter plasmid pEGFP. Nevertheless, the results of other studies demonstrated effective transfection of endothelial cells by magnetofection.^16,34,35^

The reason for the lack of magnetofection efficacy in 2H-11 cells is not known. But if we take into consideration that the main uptake mechanism for SPIONs-PAA-PEI-pDNA complexes is endocytosis,^11,36,37^ the lack of magnetofection efficacy in 2H-11 cells might be due to the size of magnetofection complexes and the size of uptake vesicles in endothelial cells. The prevailing mechanism for the uptake of macromolecules into endothelial cells is through caveolae, cell surface invaginations, measuring 50–100 nm in diameter.^38^ These endocytotic vesicles could be too small for the uptake of SPIONs-PAA-PEI-pDNA complexes, which were used in this study and measure ~200–400 nm in diameter.^11^ In the other two studies, where effective magnetofection of endothelial cells was achieved, the used complexes for magnetofection were smaller than 200 nm.^16,34,35^ Furthermore, the difference in the magnetofection efficiency among melanoma and endothelial cells could also be due to their different metabolic activity. It is well known that the uptake of nutrients through endocytosis into tumor cells is increased, due to increased needs for biosynthesis and their autonomous behavior.^39^ In a few more studies, the pronounced uptake of magnetofection complexes into tumor cells compared to normal cells was demonstrated.^11,36,40^ In contrast to magnetofection, gene electrotransfer enables plasmid DNA to enter the cells by multiple mechanisms, *i.e.*, endocytosis and electropores.^9,41,42,43^ Therefore, it is possible that the combination of both uptake mechanisms contributed to a better transfection of 2H-11 cells by electroporation.

The biological properties of melanoma cells were altered after effective silencing of *Mcam* by magnetofection or gene electrotransfer with pMCAM. Both methods of transfection were efficient in reducing proliferation and migration of melanoma cells, either B16F1 or B16F10. The obtained results were statistically significant and comparable with the results from the other studies, where antiproliferative and antimigratory effects were demonstrated after using antibodies against MCAM or small noncoding RNA molecules for *Mcam* silencing in various cell lines.^18,19,20,22–26,44,45^ In our study, we demonstrate even more pronounced inhibition of cell proliferation after *Mcam* silencing, for 79% in B16F1 and 65% in B16F10 cells. In other studies, the proliferation was reduced for 40% in human umbilical vein endothelial cells (HUVEC) after lipofection with siRNA against MCAM or even not affected in primary and metastatic melanoma cells after retroviral transfection with plasmid DNA encoding small noncoding RNA molecule against MCAM.^22,44^ On the other hand, treating cells with antibodies against MCAM reduced proliferation only in HUVEC for ~60%, while the proliferation of osteosarcoma, melanoma, hepatocarcinoma, cervix, and ovary tumor cells was not affected.^18,19,20^ Furthermore, we showed that the migration of B16F10 cells after magnetofection or gene electrotransfer with pMCAM was also effectively inhibited, for 50 or 60%, respectively. In one study, where the migration of cells was affected for 50%, gene electrotransfer was used for the delivery of siRNA against MCAM into human melanoma cells.^23^ *Mcam* silencing by siRNA after lipofection also reduced the migration of HUVEC and breast cancer cells for 70 and 40%, respectively.^22,25^ The studies, where antibodies against MCAM were used, demonstrated a reduction of migration only in HUVEC, where it was 75%.^18,45^

In addition to the antiproliferative and antimigratory effects of *Mcam* silencing, an antiangiogenic effect was also demonstrated in 2H-11 cells by a tube formation assay. The inhibitory angiogenic effect of antibodies against MCAM in HUVEC was previously demonstrated also by a tube formation assay *in vitro* and a chicken chorioallantoic membrane (CAM) assay *in vivo*.^18,19^ In our study the reduced proliferation and the ability to form capillary-like structures, as a surrogate measure of angiogenesis, were obtained only after gene electrotransfer with pMCAM, whereas magnetofection was not effective, as was expected, based on its poor transfection efficiency in 2H-11 cells and subsequent silencing efficiency.

Finally, the *in vivo* antitumor effectiveness of magnetofection and gene electrotransfer with pMCAM was demonstrated in the murine tumor model. Among the B16F1 and B16F10 tumor models, we decided on the B16F10 tumor model because it presented us a greater challenge in treatment due to its complexity and aggressiveness. A significant antitumor effect was obtained by both methods; however the effectiveness of gene electrotransfer was significantly better as measured by tumor growth delay and tumor cures. In the case of magnetofection, the tumor growth was delayed only during the time course of treatment. First, the short delay could be the consequence of low transfection efficiency, rapid proliferation of tumor cells leading to the loss of plasmid pMCAM with every cell division. Second, based on our *in vitro* data, tumor endothelial cells are probably not affected also *in vivo*, therefore the antiangiogenic component after magnetofection with pMCAM was not expressed. It seems that predominantly antiproliferative and antimigratory effect of magnetofection with pMCAM in tumor cells contributed to its antitumor effect in melanoma tumors. In contrast to magnetofection, gene electrotransfer affects both, tumor endothelial as well as tumor cells, therefore the antiangiogenic, antiproliferative and antimigratory components were expressed after gene electrotransfer of pMCAM and contributed to its greater antitumor effectiveness. Nevertheless, the induction of immune response in very immunogenic B16F10 tumors by gene electrotransfer also contributed to the observed antitumor effect after gene electrotransfer, even when pSCR was used, as we already described previously in our collaboration with Heller.^4^ In the case of magnetofection with pSCR, no antitumor effect was observed; therefore, we can assume that the obtained antitumor effect of magnetofection with pMCAM was exclusively due to the silencing of *Mcam*.

To the best of our knowledge, this is the first study to investigate the antitumor effect of gene therapy, targeting MCAM with a new approach, RNAi, in a mouse tumor model *in vivo*. So far, only two research groups have targeted MCAM in *in vivo* models. They used antibodies against MCAM and examined their effect on tumor growth, angiogenesis and metastasis formation.^18,19^ In the study, where an ABX-MA1 antibody was used, an approximately threefold reduction in tumor growth and approximately fivefold lower number of metastases in the lungs compared to the control group were demonstrated.^19^ The obtained antitumor effect was, in contrast to our predictions, not attributed to the inhibition of cell proliferation, since *in vitro* testing on the ABX-MA1 antibody failed to prove that. They have demonstrated that the antitumor and antimetastatic effects are the result of cell-cell and cell-matrix inhibition, reduction of cell invasion and tumor angiogenesis.^19^ The other *in vivo* tested antibody against MCAM was AA98. The therapy of pancreatic cancer, leiomyosarcoma and liver carcinoma in mice with AA98 resulted in the inhibition of tumor growth for 41, 50, and 72, respectively, the formation of metastasis in lungs and lymph nodes, and also a 70% reduction in the density of blood vessels. They attributed the antitumor effect, similar to us, to the inhibition of tumor angiogenesis, which was demonstrated with *in vitro* testing of HUVEC proliferation and migration, and an *in vivo* CAM assay.^18^ The results of *in vivo* studies, targeting MCAM either by antibodies against MCAM or plasmid DNA encoding shRNA against MCAM, demonstrate that the antitumor and antimetastatic effects are the outcome of several complementary effects of MCAM inactivation or *Mcam* silencing, such as the inhibition of tumor and endothelial cell proliferation, invasion, migration, and antiangiogenic effect in endothelial cells. Since MCAM is involved in the development and progression of cancer through several mechanisms, it represents a potential target in the cancer treatment with less possible development of resistance to the therapy.

To date, this was also one of the first *in vivo* studies using magnetofection for treating tumors with therapeutic plasmid DNAs. Only three other research groups had used magnetofection for the treatment of tumors *in vivo,* and demonstrated its efficiency, safety and potential use for the delivery of therapeutic genes.^11,46,47,48,49^ However, only in two studies was antitumor effect of magnetofection with therapeutic genes demonstrated. In the first study magnetofection was investigated in combination with RNAi, similar to our study. A plasmid DNA encoding shRNA against type 1 insulin-like growth factor receptor was used for magnetofection of lung adenocarcinoma in mice and resulted in a suppression of tumor growth rate for ~36% in comparison with the growth rate obtained by lipofection.^48^ In another study, they also demonstrated the antitumor effect of magnetofection with plasmid DNA encoding tumor necrosis factor-related apoptosis-inducing ligand in adenoid cystic carcinoma in mice.^49^ In our previous study, the magnetofection of TS/A tumors in mice with therapeutic plasmid DNA encoding IL-12 resulted also in a significant antitumor effect, which was comparable to gene electrotransfer.^11^

Our investigations on the use of magnetofection as a nonviral transfection method demonstrated its potential for the delivery of therapeutic plasmid DNA into the cells *in vitro* and tumors *in vivo*. Magnetofection proved to be a feasible method for noninvasive and painless gene delivery; however, a difference in effectiveness among magnetofection and gene electrotransfer was observed, indicating a need for further improvement of magnetofection transfection efficiency also in the cell type's refractory to magnetofection.

## Materials and Methods

*Cell lines and culturing.* Murine melanoma cell lines, B16F1 with low and B16F10 with high metastatic potential (American Type Culture Collection, Manassas, VA), were cultured in an advanced minimum essential medium (AMEM, Gibco by Life Technologies, Grand Island, NY), supplemented with 5% fetal bovine serum (FBS, Gibco), 10 ml/l L-glutamine (GlutaMAX, Gibco), 100 U/ml penicillin (Grünenthal, Aachen, DE) and 50 mg/ml gentamicin (Krka, Novo mesto, SI), and in a 5% CO_2_ humidified incubator at 37 °C.

A murine endothelial cell line 2H-11 (American Type Culture Collection) was cultured in an advanced Dulbecco's modified eagle medium (ADMEM, Gibco), supplemented with 5% FBS (Gibco), 10 ml/l L-glutamine (Gibco), 100 U/ml penicillin (Grünenthal), and 50 mg/ml gentamicin (Krka), and in a 5% CO_2_ humidified incubator at 37 °C.

For experiments, cells were grown as a monolayer in a 15 cm Petri dish (Techno Plastic Products, TPP, Trasadingen, CH) and maintained in a humidified atmosphere of 5% CO_2_ at 37 °C, until they reached at least 80% confluence. Then the medium was removed, the cells were washed with phosphate-buffered saline (PBS, Merck Millipore, Darmstadt, DE) and detached with 0.25% trypsin/EDTA in Hank's buffer (Gibco). For trypsin inactivation, an equal volume of AMEM or ADMEM with FBS was added, cells were then collected in a 50 ml conical falcon tube (TPP), centrifuged, counted in a hemocytometer and prepared at different densities for further experiments.

*The selection of the most effective siRNA molecule against MCAM.* A set of three, 25 nucleotides long, siRNA duplexes (Stealth RNAi siRNA, Invitrogen by Life Technologies, Carlsbad, CA), targeting different sections of coding sequences of murine MCAM mRNA, was selected using freely available BLOCK-iT RNAi Designer software (Invitrogen) (**Supplementary Table S1**). The negative control siRNA duplexes (Stealth RNAi Negative Control Duplexes, Invitrogen) were designed with the manufacturer's assurance to minimize sequence homology to any known vertebrate transcript. The siRNA duplexes were obtained as ready-annealed, purified duplexes and diluted in sterile diethylpyrocarbonate (DEPC) treated water to a concentration of 20 µmol/l.

*Lipofection of the cells with siRNA molecules.* After the cells were trypsinized, centrifuged and counted, a cell suspension in a particular antibiotics-free medium was prepared. Cells (1.4 × 10^6^) were plated on a 24-well ultra-low attachment plate (Corning, Corning, NY) in 1.5 ml of a particular antibiotics-free medium. Then complexes of siRNA duplexes and Lipofectamine RNAiMAX (Invitrogen) were prepared as follows: 5 µl of 20 µmol/l siRNA was diluted in 500 μl antibiotics-free Opti-MEM I medium (Gibco) to which 5 µl of Lipofectamine RNAiMAX was added. After 15 minutes incubation at room temperature the complexes were added to cells in each well. Cells were incubated at 37 °C in a 5% CO_2_ humidified incubator for 5 hours and then plated on 10 cm or 15 cm Petri dishes (TPP) for further qRT-PCR analysis, flow cytometry, proliferation, clonogenic, wound healing, and tube formation assays.

*The construction of plasmid DNA encoding shRNA against MCAM.* The plasmid DNA encoding shRNA against MCAM (pMCAM) was constructed based on the nucleotide sequence of the siRNA molecule against MCAM, which was determined as the most effective in the *in vitro* experiments. First, two complementary single-stranded DNA oligonucleotides (ss oligo) were purchased from Invitrogene; one encoding shRNA against MCAM (more precisely; containing a 4-nucleotide 5′ overhang, a 26-nucleotide sequence derived from the target gene, a short 4-nucleotide spacer (*i.e.*, loop) and a 26-nucleotide sequence that is the reverse complement of the initial target sequence) and the other its complement. Then standard molecular biology techniques of annealing, ligation and transformation into competent *E. coli* TOP10 (BLOCK-iT U6 RNA Entry Vector Kit; Invitrogen) were used. The expression of the shRNA molecule from the plasmid DNA in the cells was controlled by the human U6 promoter. The plasmid DNA was amplified in *E. coli* (TOP10; Invitrogen) and isolated using a NoEndo JETSTAR ENDOTOXIN-FREE MEGA/GIGA Kit (Genomed, Löhne, DE) according to the manufacturer's protocol. The quantity of isolated plasmid DNA was determined by a spectrophotometer at 260 nm (Epoch Microplate Spectrophotometer, Take3 Micro-Volume Plate, BioTek, Bad Friedrichshall, DE) and the quality by measuring the ratio of absorbance at A_260 nm/280 nm_ and by agarose gel electrophoresis. The working concentration of 1 mg/ml was prepared with endotoxin-free water.

In addition to the preparation of pMCAM, a plasmid DNA encoding shRNA with scrambled nucleotide sequence of the most effective siRNA molecule against MCAM (pSCR) was also constructed. It was designed with the assurance of having no sequence homology to any known vertebrate transcript. Both plasmids were than sequenced to confirm the presence, correct orientation and sequence of the double-stranded DNA oligonucleotide (ds oligo) insert.

*In vitro transfection with plasmid DNA encoding shRNA against MCAM*

*Magnetofection.* After the cells were trypsinized, centrifuged and counted, a cell suspension in the particular medium was prepared. Then the cells (2.5 × 10^4^ B16F1, 2.5 × 10^4^ B16F10, or 2.0 × 10^3^ 2H-11 cells per well) were plated on a clear-bottomed 24-well test plate (TPP) in 1 ml of the particular medium for 24 hours. Before magnetofection superparamagnetic iron-oxide nanoparticles (SPIONs) were synthesized (**Supplementary Figures S9 and S10**) and stabilized with polyacrilyc acid (PAA), functionalized with polyethlenimine (PEI) and coupled with plasmid DNA (pDNA) as described previously.^12^ Immediately prior to magnetofection, SPIONs-PAA were functionalized with PEI and pDNA was bound to SPIONs-PAA-PEI complexes by mixing 21.2 µl of SPIONs-PAA-PEI with 2 µl of pDNA (2 µg). Therefore, the final mass ratio of SPIONs-PAA, PEI and pDNA was 0.6:1:1. Then magnetofection was performed by the addition of SPIONs-PAA-PEI-pDNA complexes to the cells and the plate was immediately placed on an array of Neodymium-Iron-Boron permanent magnets (Nd-Fe-B magnets with surface magnetic flux density of 403 mT and magnetic gradient of 38 T/m; Supermagnete, Uster, CH) for 15 minutes. Thereafter, the cells were incubated for 48 hours at 37 °C in a 5% CO_2_ humidified atmosphere for further qRT-PCR analysis, flow cytometry, proliferation, wound healing and tube formation assays.

In addition to magnetofection with therapeutic plasmid DNA (MF + pMCAM), there were also five control groups: untreated cells (B16F1, B16F10, or 2H-11), cells treated with control plasmid DNA (pSCR), cells treated with therapeutic plasmid DNA (pMCAM), cells treated with SPIONs-PAA-PEI complexes without plasmid DNA and exposed to external magnets (MF) and magnetofection with control plasmid (MF + pSCR).

Additionally, to determine transfection efficiency, the magnetofection of cells was performed also with plasmid DNA encoding enhanced green fluorescent protein (pEGFP, BD Biosciences Clontech, Palo Alto, CA) and the images of transfected cells were captured 48 hours after transfection with a digital camera (DP72, Olympus, Hamburg, DE) connected to an inverted fluorescence microscope (IX70, Olympus) with appropriate excitation (460–490 nm) and emission filters (515 nm long pass).

*Gene electrotransfer.* After the cells were trypsinized and centrifuged, they were washed two times in an ice-cold buffer (125 mmol/l sucrose; 10 mmol/l K_2_HPO_4_; 2.5 mmol/l KH_2_PO_4_; 2 mmol/l MgCl_2_ × 6H_2_0). For gene electrotransfer a cell suspension in an ice-cold buffer with a density of 25 × 10^6^ cells/ml was prepared. To 44 µl of prepared cell suspension 11 µl of plasmid DNA was added (11 µg). Then 50 µl of the resulting mixture (1 × 10^6^ cells) was pipetted between two stainless-steel parallel plate electrodes with a 2 mm gap in between. Eight square wave electric pulses (EP), with a voltage-to-distance ratio of 600 V/cm, pulse duration of 5 ms and frequency of 1 Hz were generated by an electric pulse generator GT-01 (Faculty of Electrical Engineering, University of Ljubljana, Ljubljana, SI).^50^ After the application of electric pulses, the cells were incubated for 5 minutes with 100 µl of FBS and then plated in the particular medium for further qRT-PCR analysis, flow cytometry, proliferation, wound healing, and tube formation assays.

In addition to gene electrotransfer of therapeutic plasmid DNA (EP + pMCAM), there were also five control groups: untreated cells (B16F1, B16F10, or 2H-11), cells treated with control plasmid DNA (pSCR), cells treated with therapeutic plasmid DNA (pMCAM), cells exposed to electric pulses without plasmid DNA (EP) and gene electrotransfer of control plasmid (EP + pSCR).

Additionally, to determine transfection efficiency, the gene electrotransfer of cells was performed also with pEGFP (BD Biosciences Clontech) and the images of transfected cells were captured 48 hours after transfection with a digital camera (DP72, Olympus) connected to an inverted fluorescence microscope (IX70, Olympus) with appropriate excitation (460–490 nm) and emission filters (515 nm long pass).

*In vitro experiments after transfection with siRNA molecules and plasmid DNA encoding shRNA against MCAM*

*Total RNA extraction and qRT-PCR analysis.* To determine MCAM expression at the mRNA level 48 hours after the transfection of cells *in vitro*, total RNA extraction and qRT-PCR analysis were performed. Cells were trypsinized and then centrifuged. Afterwards the total RNA was extracted from the cells with a TRIzol Plus RNA Purification System (Invitrogen) according to the manufacturer's instructions. Concentrations of RNA were quantified by a spectrophotometer at 260 nm (Epoch). The purity of RNA was determined by measuring the ratio of absorbance at A_260 nm/280 nm_. One μg of total RNA was reverse transcribed into complementary DNA (cDNA) using a SuperScript VILO cDNA Synthesis Kit (Invitrogen) according to the manufacturer's instructions. After reverse transcription, 10× diluted and 100× diluted mixtures were used as a template for the qRT-PCR using a TaqMan Gene Expression Master Mix (Applied Biosystems, Carlsbad, CA) and TaqMan Gene Expression Assay (Applied Biosystems). The Taqman Gene Expression Assay contained a pair of primers and TaqMan probes to amplify the fragment of murine Mcam cDNA (Mm00522397_m1). As an internal control TaqMan probes were used to amplify murine 18S ribosomal RNA (Mm03928990_g1). The qRT-PCR analysis was performed on 7300 System (Applied Biosystems) as follows: activation of Uracil-DNA Glycosylase (2 minutes at 50 °C), hot start activation of AmpliTaq Gold Enzyme (10 minutes at 95 °C), 45 cycles of denaturation (15 seconds at 95 °C), annealing and extension (1 minute at 60 °C). The qRT-PCR products were analyzed using 7300 System SDS software (Applied Biosystems). The levels of MCAM mRNA expression in murine melanoma and endothelial cell lines were presented as the threshold cycle value (Ct). The values of MCAM mRNA level for each experimental group were normalized to the values obtained for the reference genes from the cells treated with Lipofectamine only (after lipofection with siRNA molecules) or untreated cells (after magnetofection or gene electrotransfer with plasmid DNA).

*Flow cytometry.* For the quantification of MCAM protein level, an immunofluorescence staining of cells and a subsequent flow cytometry analysis was performed. The cells were trypsinized 48 hours after transfection and 1 × 10^6^ cells were resuspended in PBS. Cells were fixed in 4% paraformaldehyde (Paraformaldehyde, Alfa Aesar, A Johnson Matthey Company, Ward Hill, MA) for 15 minutes and permeabilized with 0.5% Tween 20 (TWEEN 20, Sigma-Aldrich, Steinheim, DE) for 10 minutes. After permeabilization the cells were incubated with 10% donkey serum (Sigma-Aldrich) diluted in PBS for 30 minutes at room temperature. Then the serum was removed and the primary goat anti-mouse polyclonal antibodies (Mel-CAM (C-20): sc-18942, 1:50, Santa Cruz Biotechnology, Santa Cruz, CA) were added to the cells for overnight incubation at 4 °C. The next day, the primary goat anti-mouse polyclonal antibodies were removed and the cells further incubated with fluorescein isothiocyanate-conjugated (FITC) donkey anti-goat secondary antibodies (FITC-AffiniPure F(ab')2 Frag Donkey Anti-Goat IgG, 1:100, Jackson ImmunoResearch Laboratories, West Grove, PA) for 1 hour. Between each step, the cells were washed three times with PBS.

The measurements were performed on at least 200,000 cells per sample using a FACSCanto II flow cytometer (BD Biosciences, San Jose, CA). A 488 nm laser (air-cooled, 20 mW solid state) and 530/30 nm band-pass filter were used for the excitation and detection of FITC fluorescence, respectively. First, the viable cell population was gated from the biparametric logarithmic plot, defined by forward and side scatter, to eliminate debris. Second, the histogram of gated cells against their fluorescence intensity was recorded. The median fluorescence intensity of the gated cells was determined for each experimental group (software: BD FACSDiva V6.1.2). The median fluorescence intensity of the cells for each experimental group was normalized to the median fluorescence intensity of the cells treated with Lipofectamine only (after lipofection with siRNA molecules) or untreated cells (after magnetofection or gene electrotransfer with plasmid DNA).

*Proliferation assay.* After *in vitro* transfection 2 × 10^2^ B16F1, B16F10, or 2H-11 cells per well were plated on 96-well plates (Corning) in 0.1 ml of the particular medium, containing FBS and antibiotics, for a proliferation assay. Cells were incubated at 37 °C in a 5% CO_2_ humidified incubator. Cell viability was measured every second day with a Presto Blue assay (Invitrogen). 10 µl of Presto Blue reagent was added to each well and fluorescence intensity (at 535–560 nm excitation wavelength and 590–615 nm emission wavelength) in each well was measured with a microplate reader (Infinite 200, Tecan, Männedorf, CH) after 1.5 hours incubation at 37 °C in a 5% CO_2_ humidified incubator. The proliferation curve of each experimental group was normalized to day 0.

*Wound healing assay.* To determine the effect of *Mcam* silencing on the migratory potential of B16F1 and B16F10 cells a wound healing assay was performed. The cells (2.5 × 10^4^ per well) were plated 24 hours after transfection on a 24-well plate with silicone inserts, which formed a 500 µm ± 50 µm cell-free gap after removal (24 Culture-Inserts, Ibidi, Munich, DE). After 24 hours incubation at 37 °C and 5% CO_2_ a confluent cell monolayer formed and the Culture-Inserts were removed using sterile tweezers. Each well was then filled with 1 ml of the particular medium containing FBS and antibiotics. The images of the wound in each tested group were captured with a digital camera (DP72, Olympus) connected to an inverted fluorescent microscope (IX70, Olympus) at the 0 hour time point and every next 2 hours until the wound was closed. At all-time points the cell-free area was quantified in a FIJI image analysis program^51^ and from the obtained values a kinetic analysis was made. The obtained cell migration rate of each experimental group was normalized to the rate of migration of cells treated with Lipofectamine only (after lipofection with siRNA molecules) or untreated cells (after magnetofection or gene electrotransfer with plasmid DNA).

*Tube formation assay.* To determine the effect of *Mcam* silencing on the ability of 2H-11 cells to form capillary-like structures, a tube formation assay was performed. The cells (1.5 × 10^4^ per well) were plated 48 hours after transfection on a µ-Slide Angiogenesis (Ibidi) covered with a BD Matrigel Basement Membrane Matrix, Phenol Red Free (BD Biosciences) and incubated for 2.5 hours until the formation of tubular complexes. One tubular complex is defined as all connected capillary-like structures. The tubular complexes were stained with Calcein AM (Sigma-Aldrich). Images were captured with a digital camera (DP72, Olympus) connected to an inverted fluorescent microscope (IX70, Olympus) with appropriate excitation (460–490 nm) and emission filters (515 nm long pass). The AxioVision program (Carl Zeiss Microscopy GmbH, Jena, DE) was used to convert raw images into binary images of the formed tubular complexes, which were quantified with the AngioQuant image analysis program.^52^ The total length and size (area) of the tubular complexes, the total number of junctions and the number of complexes (the more organized a tubular complex is, the lower the number of complexes) were quantified. Determined parameters of the tube formation assay for each experimental group were normalized to determined parameters of the cells treated with Lipofectamine only (after lipofection with siRNA molecules) or untreated cells (after magnetofection or gene electrotransfer with plasmid DNA).

*In vivo experiments with plasmid DNA encoding shRNA against MCAM*

*Animals.* Female C57Bl/6 mice were purchased from Harlan Laboratories (Udine, IT) and subjected to an adaptation period of 2 weeks. Mice were housed in specific pathogen-free conditions at a temperature of 20–24 °C, relative humidity 55 ± 10% and a 12 hours light/dark cycle. Food and water were provided ad libitum. All procedures were performed in compliance with the guidelines for animal experiments of the EU directive (2010/63/EU) and permission from the Veterinary Administration of the Ministry of Agriculture and the Environment of the Republic of Slovenia (permission no. 34401–4/2012/2).

*Magnetofection and gene electrotransfer of tumors.* Animals were 10 weeks old when B16F10 tumors were induced. A suspension of 1 × 10^6^ B16F10 cells, prepared from a cell culture *in vitro* in 0.1 ml of physiological solution, was subcutaneously injected into the shaved right flank of the mice. When the tumors reached ~40 mm^3^ (in 4–5 days after subcutaneous injection of cells), the animals were randomly divided into nine experimental groups, consisting of six animals per group and subjected to specific experimental protocols for 3 consecutive days.

The first group (Control) of animals was injected intratumorally (i.t.) with 40 µl of endotoxin-free water. The second (pSCR) and third groups (pMCAM) of animals were injected i.t. with 15 µg of pSCR or pMCAM in 40 µl of endotoxin-free water. The fourth group (MF) of animals was injected i.t. with 40 µl of SPIONs-PAA-PEI complexes at a mass ratio of 0.6:1. The fifth (MF + pSCR) and sixth groups (MF + pMCAM) of animals were injected i.t. with 15 µg of pSCR or pMCAM prepared in 40 µl of SPIONs-PAA-PEI-pDNA complexes at a mass ratio of 0.6:1:1. The i.t. injections were performed slowly and lasted ~5 seconds (8 µl/second). Thereafter, Nd-Fe-B magnets (the same magnets as those used for *in vitro* experiments) were placed above the tumors of the fourth, fifth and sixth groups and fixed with an adhesive tape (Micropore plaster, 25 mm × 9.1 m, Tosama, Vir, SI) for 30 minutes. The animals were kept under inhalation anesthesia with 1.5% isoflurane (Izofluran Torrex para 250 ml, Chiesi Slovenia, Ljubljana, SI) with an oxygen flow of 1 l/minute during i.t. injection for precise injection into small tumors, and during the exposure of the tumors to the magnet.

The seventh group (EP) of animals was injected i.t. with 40 µl of endotoxin-free water. The eighth (EP + pSCR) and ninth groups (EP + pMCAM) of animals were injected i.t. with 15 µg of pSCR or pMCAM in 40 µl of endotoxin-free water. The i.t. injections in the seventh, eighth and ninth groups were performed on anesthetized animals for precise injection into small tumors. The i.t. injections were performed slowly and lasted ~5 seconds (8 µl/second). Ten minutes after i.t. injection, electric pulses (eight square wave pulses, delivered in two sets of four pulses in perpendicular directions, at a frequency of 1 Hz, amplitude over distance ratio of 600 V/cm and 5 ms duration through two parallel stainless steel electrodes with a 4 or 6 mm gap) generated by electric pulse generator ELECTRO CELL B10 (Betatech, L'Union, Saint-Orens-de-Gameville, FR) were applied to the tumors of the seventh, eighth and ninth groups.^50^

Tumor growth and regression was monitored until the tumors reached between 300 and 350 mm^3^ and then the animals were euthanized. The tumors were measured in three orthogonal diameters with a Vernier caliper, and their volumes were calculated using the equation *V* = a × b × c × π/6. The tumor growth delay for each experimental group was calculated as the difference in tumor doubling times of experimental and control group. Tumor doubling time is the number of days in which the initial tumor volume (40–50 mm^3^) doubles. Mice that remained tumor-free for 100 days were termed as cured (complete response). Animal weight was used as a general index of systemic toxicity.

*Statistical analyses.* All quantitative data are presented as mean (AM) ± SEM. The data were tested beforehand for normality of distribution using the Shapiro-Wilk test and statistically processed by SigmaPlot statistical software (version 12.0, Systat Software, London, UK). Differences between the experimental groups were evaluated by one-way analysis of variance followed by the Holm–Sidak test for multiple comparisons. For the comparison of two experimental groups a Student's *t*-test was used. Alpha level was set to 0.05. A probability level of *P* < 0.05 was considered to be statistically significant.

**SUPPLEMENTARY MATERIAL** **Figure S1.** The MCAM mRNA level after lipofection with selected siRNA molecules against MCAM. **Figure S2.** The MCAM protein level after lipofection with selected siRNA molecules against MCAM. **Figure S3.** The proliferation of cells after lipofection with selected siRNA molecules against MCAM. **Figure S4.** The survival of cells after lipofection with selected siRNA molecules against MCAM. **Figure S5.** The migration of B16F10 cells after lipofection with selected siRNA molecules against MCAM. **Figure S6.** The B16F10 cell migration rate after lipofection with selected siRNA molecules against MCAM. **Figure S7.** The images of the tube formation assay obtained 48 hours after lipofection with selected siRNA molecules against MCAM. **Figure S8.** The tubular properties (total length and size, number of junctions) and the number of complexes obtained 48 hours after lipofection with selected siRNA molecules against MCAM. **Figure S9.** Transmission electron micrograph (TEM) of spherical, crystalline and slightly agglomerated SPIONs. **Figure S10.** X-ray diffractograms (XRD) of SPIONs. **Table S1.** The origin, nucleotide sequences and the targeted nucleotide section of murine MCAM mRNA of selected siRNA molecules.

## Figures and Tables

**Table 1:**
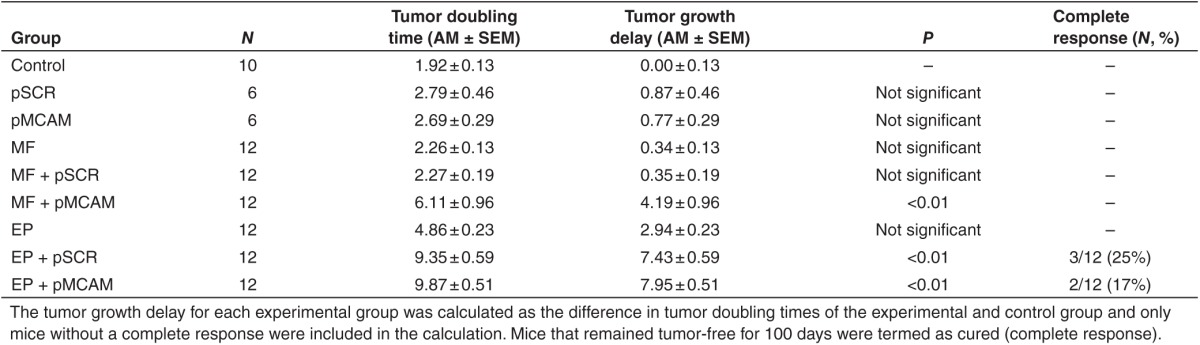
The effect of magnetofection (MF) and gene electrotransfer (EP) with pMCAM on B16F10 murine melanoma tumors
