# Supplementary material for: Mcam Silencing With RNA Interference Using Magnetofection has Antitumor Effect in Murine Melanoma
Source: Mol Ther Nucleic Acids. 2014 Oct 28;3(10):e205–. doi: 10.1038/mtna.2014.56 (PMC4217080; doi:10.1038/mtna.2014.56)
Supplement: Supplementary Information [file mtna201456x1.doc]

SUPPLEMENT

RESULTS WITH siRNA MOLECULES AGAINST MCAM

1. *All tested siRNA molecules silenced Mcam gene expression and decreased MCAM protein level in the cells*

To determine if any of the selected siRNA molecules against MCAM, targeting different coding sequences of murine *Mcam* gene, reduce the expression of MCAM at the mRNA level, the total mRNA was isolated from 2H-11, B16F1 and B16F10 cells 48 h after lipofection and a qRT-PCR analysis was performed. All tested siRNA molecules against MCAM statistically significantly decreased the expression of MCAM at the mRNA level in all treated cell lines by (**Figure S1**). The level of MCAM mRNA was reduced up to 74% in 2H-11, 70% in B16F1 and 76% in B16F10 cells. There was no statistically significant difference in the effectiveness among all tested siRNA molecules against MCAM.

Further, we were interested whether siRNA molecules against MCAM similarly inhibited the expression of MCAM at the protein level. Flow cytometry analysis on the immunocytochemicaly stained samples, in order to detect MCAM protein expression in 2H-11, B16F1 and B16F10 cells 48 h after lipofection was performed. All tested siRNA molecules against MCAM statistically significantly decreased the expression of MCAM at the protein level in all treated cell lines (**Figure S2**). The level of MCAM protein was reduced up to 23% in 2H-11, 62% in B16F1 and 39% in B16F10 cells. There was no statistically significant difference in the effectiveness among all tested siRNA molecules against MCAM.

These results indicate that all selected siRNA molecules against MCAM selectively block MCAM expression at both mRNA and protein level.


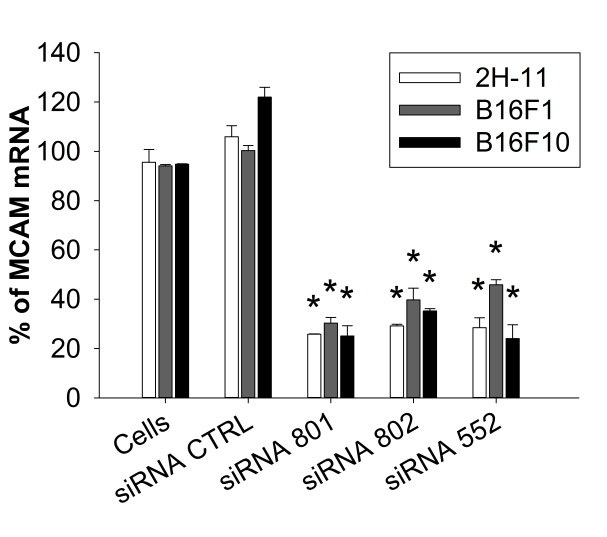


**Figure S1 The MCAM mRNA level after lipofection with selected siRNA molecules against MCAM.** Bars represent AM ± SEM of the percentage of MCAM mRNA (N=2). Asterisks indicate statistically significant differences between three control groups (cells, cells treated with Lipofectamine only, siRNA CTRL) and the group compared (*p<0.05). All data are normalized to MCAM mRNA in the cells treated with Lipofectamine only.


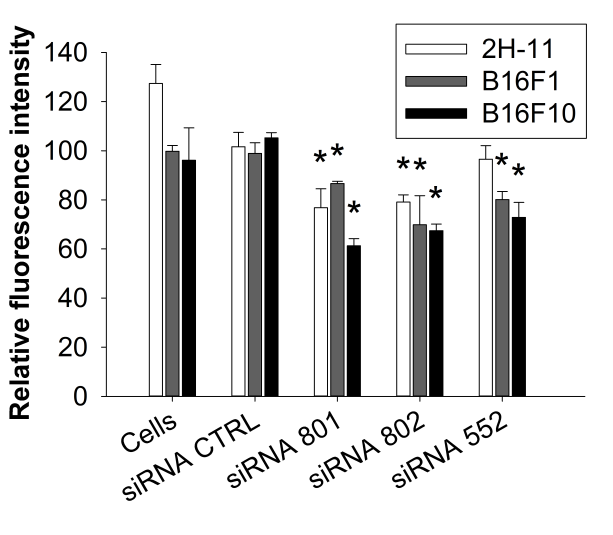


**Figure S2 The MCAM protein level after lipofection with selected siRNA molecules against MCAM.** Bars represent AM ± SEM of the relative fluorescence intensity (N=3). Asterisks indicate statistically significant differences between three control groups (cells, cells treated with Lipofectamine only, siRNA CTRL) and the group compared (*p<0.05). All data are normalized to fluorescence intensity of the cells labeled with FITC-MCAM antibodies and treated with Lipofectamine only.

1. *Silencing of Mcam has a minor effect on the cell growth and survival*

Since we demonstrated a decrease in the level of MCAM mRNA and protein after lipofection with different siRNA molecules against MCAM, we further examined the possible cytotoxic effect of *Mcam* silencing in 2H-11, B16F1 and B16F10 cells. A minor inhibition of proliferation was detected after transfection with siRNA 552 at day 2 and 4 in 2H-11 and B16F1 cells, but only at day 4 in B16F10 cells (**Figure S3**). The survival of B16F1 and B16F10 cells determined by colony forming assay was decreased for 18% and 26%, respectively by siRNA 801 and for 28% in B16F1 cells by siRNA 552 (**Figure S4**).


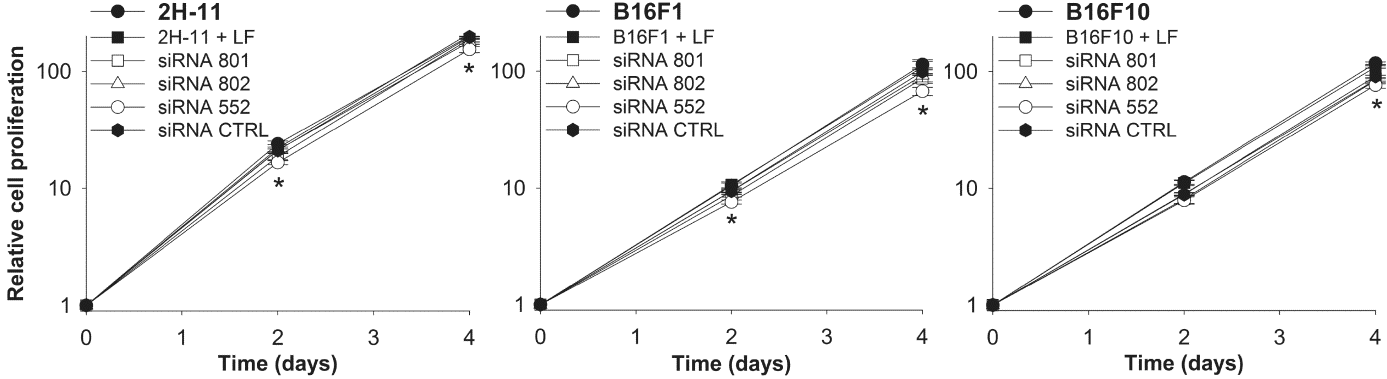


**Figure S3 The proliferation of cells after lipofection with selected siRNA molecules against MCAM.** The proliferation of 2H-11, B16F1 and B16F10 cells was monitored for four days. All data for cell proliferation curves are expressed as AM ± SEM (N=24). Asterisks indicate statistically significant differences between three control groups (cells, cells treated with Lipofectamine (LF) only, siRNA CTRL) and the group compared (*p<0.05). All data are normalized to day 0.


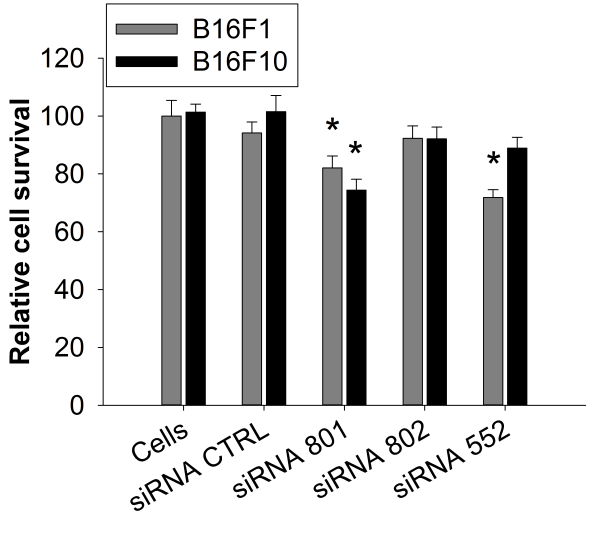


**Figure S4 The survival of cells after lipofection with selected siRNA molecules against MCAM.** Bars represent AM ± SEM of the relative cell survival (N=2). Asterisks indicate statistically significant differences between three control groups (cells, cells treated with Lipofectamine only, siRNA CTRL) and the group compared (*p<0.05). All data are normalized to the survival of cells treated with Lipofectamine only.

1. *The migration of B16F10 cells was significantly slower after lipofection with siRNA molecules against MCAM*

Additionally we examined the effect of *Mcam* silencing on biological properties of B16F10 cells. We examined the antimigratory effect of selected siRNA molecules against MCAM 48 h after lipofection with the wound healing assay. Antimigratory effect could not be determined in B16F1 cells, because they started to migrate barely after 30 h, and during this time they grew in the multilayer and, consequently died (data not shown).

The healing of the wound or migration of the cells in the cell-free area is shown in **Figure S5**. Already from the images, a difference in the cell migration rate among the tested groups can be observed. There was no cell-free area left after 15 h in the group with untreated cells and cells treated with Lipofectamine only. However, after treating cells with siRNA 801 cell-free area contained only few migratory cells.

The kinetic analysis of cell migration confirmed that only silencing of *Mcam* with siRNA 801 molecule statistically significantly reduced the cell migration rate, namely cells migrated 1.5-fold slower than in the control groups (**Figure S6**). The other two siRNA molecules against MCAM had no effect on cell migration.

The wound healing assay confirmed that even though all three selected siRNA molecules against MCAM effectively blocked MCAM expression on the mRNA and the protein level, it does not necessarily mean that this would have an effect also on the biological properties of the cells. However, only silencing of *Mcam* with siRNA 801 molecule had antimigratory effect in B16F10 cells.


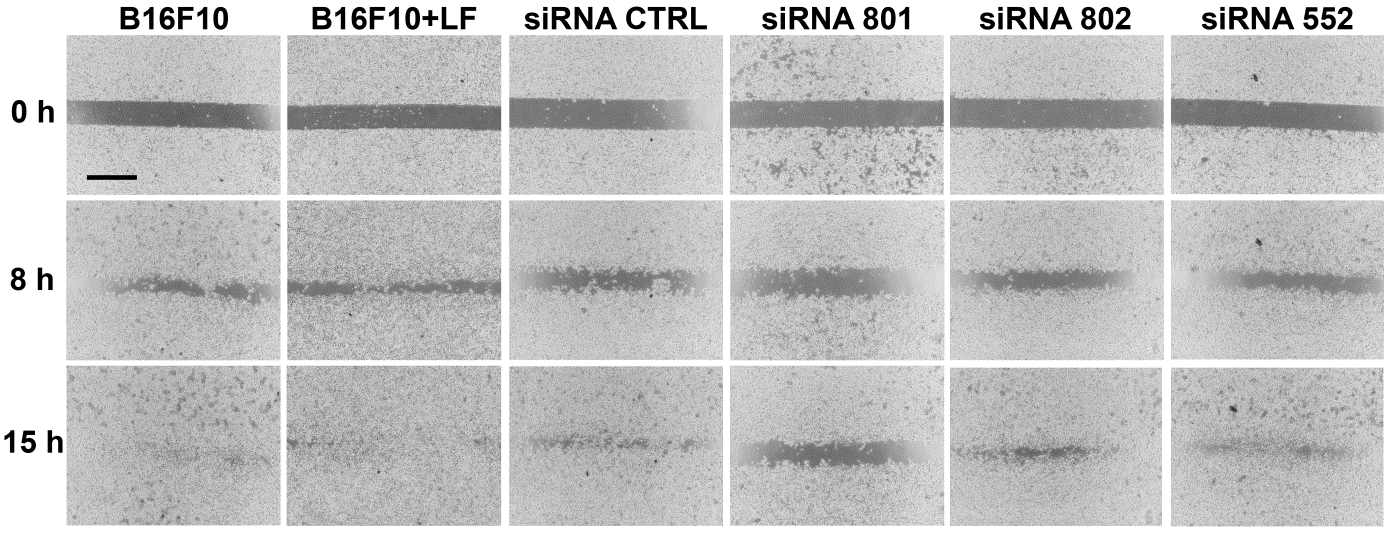


**Figure S5** **The migration of B16F10 cells after lipofection with selected siRNA molecules against MCAM.** The images were taken under visible light, 4x magnification and at 0 h (first line), 8 h (second line) and 15 h (third line) after the removal of silicone cell-separator. LF is the abbreviation for Lipofectamine. Scale bar, 500 µm.


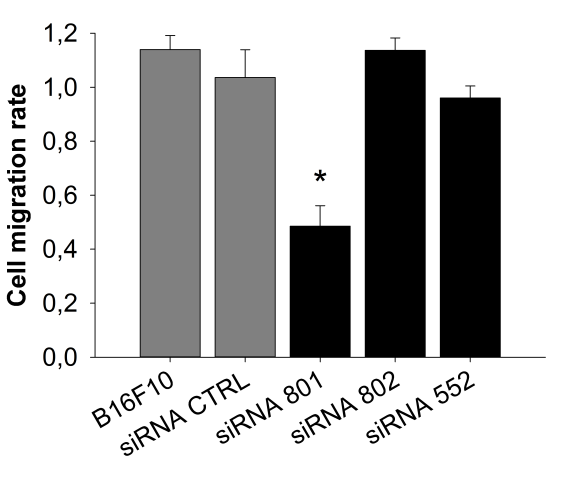


**Figure S6 The B16F10 cell migration rate after lipofection with selected siRNA molecules against MCAM.** Bars represent AM ± SEM of the cell migration rate (N=5-9). Asterisks indicate statistically significant differences between three control groups (cells, cells treated with Lipofectamine only, siRNA CTRL) and the group compared (*p<0.05). All data are normalized to the cell migration rate of cells treated with Lipofectamine only.

1. *The ability of 2H-11 cells to form capillary like structures was slightly affected after lipofection with siRNA molecules against MCAM*

Since MCAM is known to be involved in tumor angiogenesis, we have investigated the antiangiogenic potential of the selected siRNA molecules against MCAM in 2H-11 endothelial cells with the tube formation assay performed 48 h after lipofection.

2H-11 cells formed capillary like structures (tubular complex) 2.5 h after seeding on Matrigel. Already from the images, a slight difference in the ability of forming capillary like structures among the cells treated with siRNA 801 or siRNA 802 molecules against MCAM and control groups can be observed (**Figure S7**). The formed capillary like structures were smaller, unusual shape and there were also numerous separate cells excluded from formed capillary like structures. The siRNA 552 as well as siRNA CTRL had no apparent effect on the ability of 2H-11 cells to form capillary like structures.

Analysis of binary masks confirmed that after transfection with siRNA molecules against MCAM the total size of formed capillary like structures was statistically significantly reduced for 20% and the number of formed separate complexes increased for approx. 3 to 4.5-fold (**Figure S8a**). From all tested siRNA molecules, siRNA 801 affected both, the total size and the number of complexes, while siRNA 802 affected only the number of complexes (**Figure S8b**). The siRNA 552 as well as siRNA CTRL had no significant effect on the quantified properties of formed capillary like structures.

The tube formation assay showed that silencing of *Mcam* with siRNA 801 had a minor antiangiogenic effect in 2H-11 cells. The siRNA 801 molecule outperformed the other two tested siRNA molecules in reduction of the size of formed capillary like structures and in increasing the number of formed separate complexes.


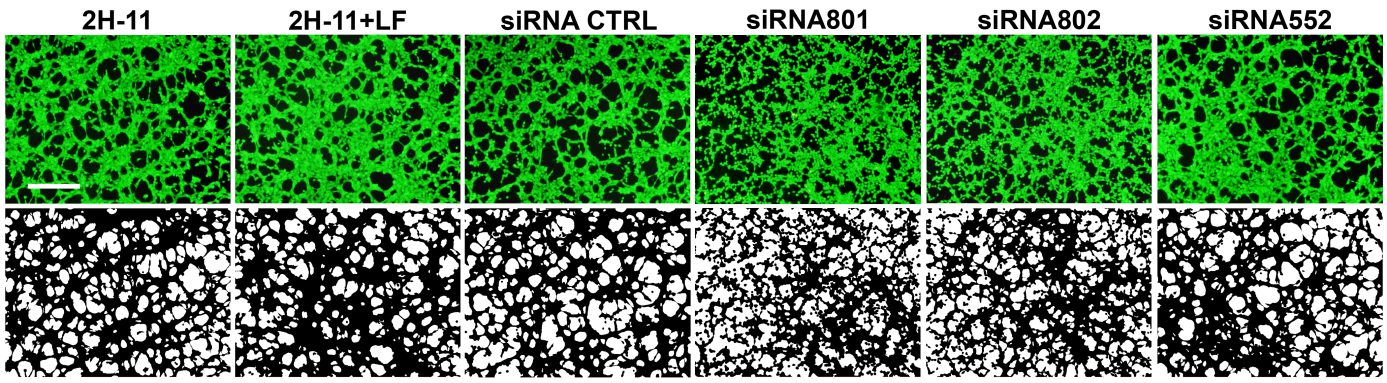


**Figure S7 The images of the tube formation assay obtained 48 h after lipofection with selected siRNA molecules against MCAM.** The images in the first row were taken under fluorescent light (excitation 460 - 490 nm and emission filters 515 nm long pass) and 4x magnification. The second row represents the binary masks, which were made from raw images for the quantification of tubular properties. LF is the abbreviation for Lipofectamine. Scale bar, 500 µm.


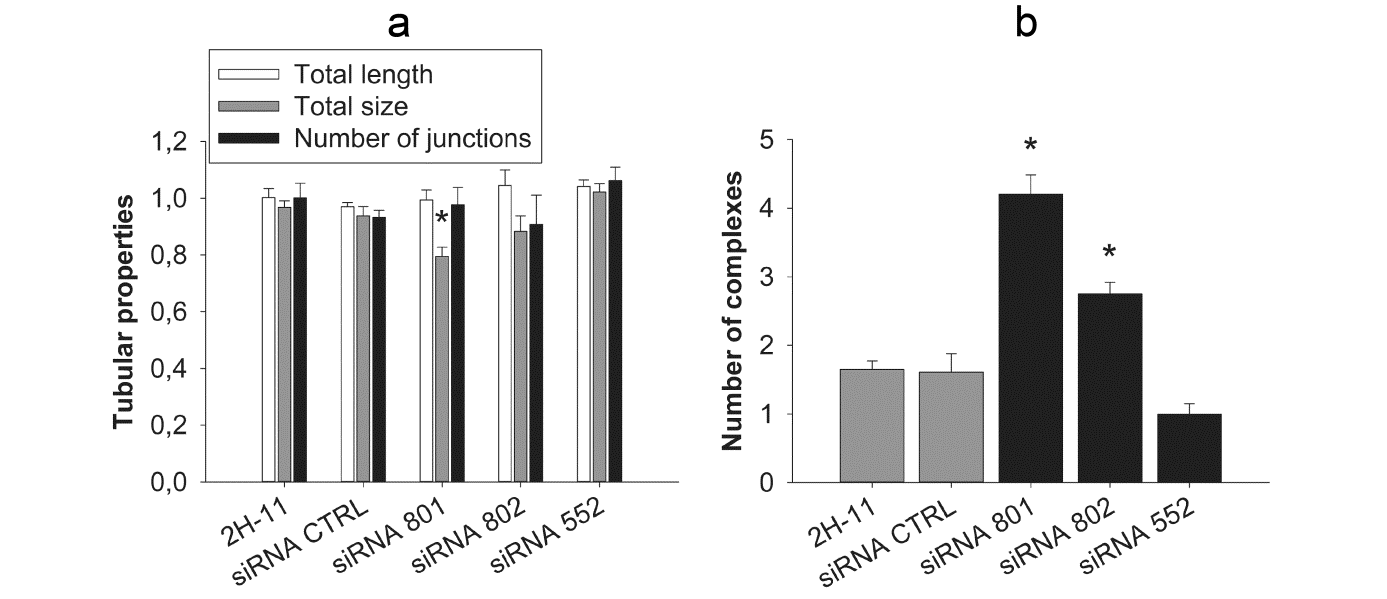


**Figure S8 The tubular properties (total length and size, number of junctions) and the number of complexes obtained 48 h after lipofection with selected siRNA molecules against MCAM.** Bars represent AM ± SEM of (**a**) the tubular properties (N=11-15) or (**b**) the number of complexes (N=10-13). Asterisks indicate statistically significant differences between three control groups (cells, cells treated with Lipofectamine only, siRNA CTRL) and the group compared (*p<0.05). All data are normalized to the tubular properties or the number of complexes of cells treated with Lipofectamine only.

**MATERIAL AND METHODS**

**Table S1** **The origin, nucleotide sequences and the targeted nucleotide section of murine MCAM mRNA of selected siRNA molecules**

| **siRNA against MCAM** | **Origin** | **Sense strand (5’-3’)** | **Antisense strand (5’-3’)** | **Targeted nucleotide section of MCAM mRNA** |
| --- | --- | --- | --- | --- |
| siRNA 801 | murine | CCA UUG UUC UGA AGC UGG UCA CUU U | AAA GUG ACC AGC UUC AGA ACA AUG G | 1570-1594 |
| siRNA 802 | murine | CCA AAC UGG UGU GCG UCU UCU UGU U | AAC AAG AAG ACG CAC ACC AGU UUG G | 43-67 |
| siRNA 552 | murine | CCA CAG ACA GUA GUG AGC ACC UUG A | UCA AGG UGC UCA CUA CUG UCU GUG G | 1467-1491 |

1. *Lipofection of the cells with siRNA molecules*

Cells were trypsinized, centrifuged, counted and prepared in a particular antibiotics-free medium. Cells (1.4 x 10^6^) were plated on 24-well ultra-low attachment plate (Corning Inc., Corning, NY) in 1.5 ml of particular antibiotics-free medium. Then complexes of siRNA duplexes and Lipofectamine RNAiMAX (Invitrogen by Life Technologies, Carlsbad, CA) were prepared as follows: 5 µl of 20 µM siRNA was diluted in 500 μl antibiotics-free Opti-MEM I medium (Gibco by Life Technologies, Grand Island, NY) to which 5 µl of Lipofectamine RNAiMAX was added. After 15 min incubation at room temperature the complexes were added to cells in each well. Cells were incubated at 37°C in a 5% CO_2_ humidified incubator for 5 h and then plated on 10 cm or 15 cm Petri dishes (Techno Plastic Products, TPP, Trasadingen, CH) for further assays: qRT-PCR analysis, flow cytometry, proliferation, clonogenic, wound healing and tube formation assays.

1. *Clonogenic assay*

After *in vitro* lipofection 2 x 10^2^ B16F1 or B16F10 cells were plated on 5 cm Petri dish (TPP) in 4 ml of particular medium containing fetal bovine serum and antibiotics. When the colonies formed (approx. 7 days after plating), they were fixed, stained with crystal violet (Crystal Violet solution, Sigma-Aldrich, Steinheim, DE) and counted. The colonies containing less than 50 cells were disregarded. The cell survival for each experimental group was normalized to the survival of cells treated with Lipofectamine only.

1. *Magnetofection*


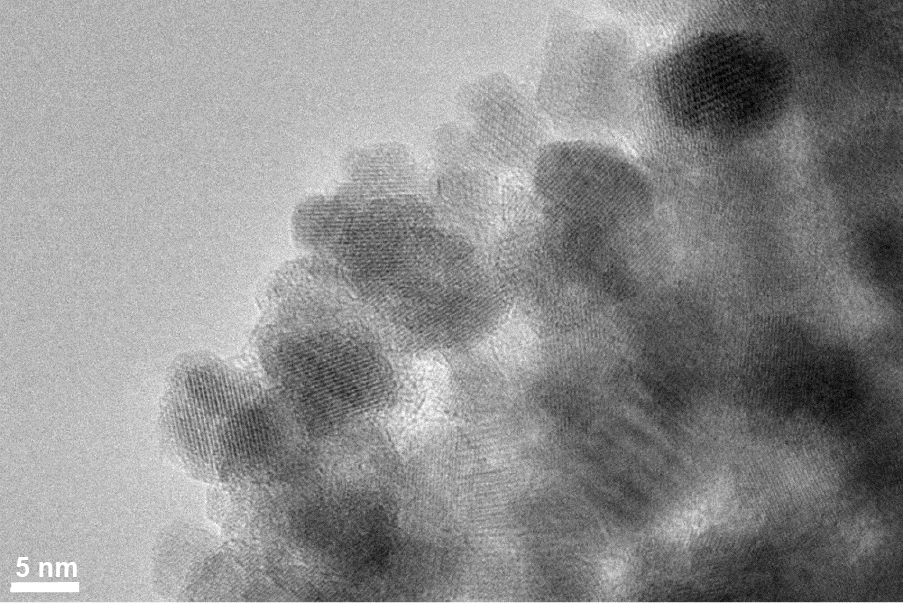


Figure S10 Transmission electron micrograph (TEM) of spherical, crystalline and slightly agglomerated SPIONs.


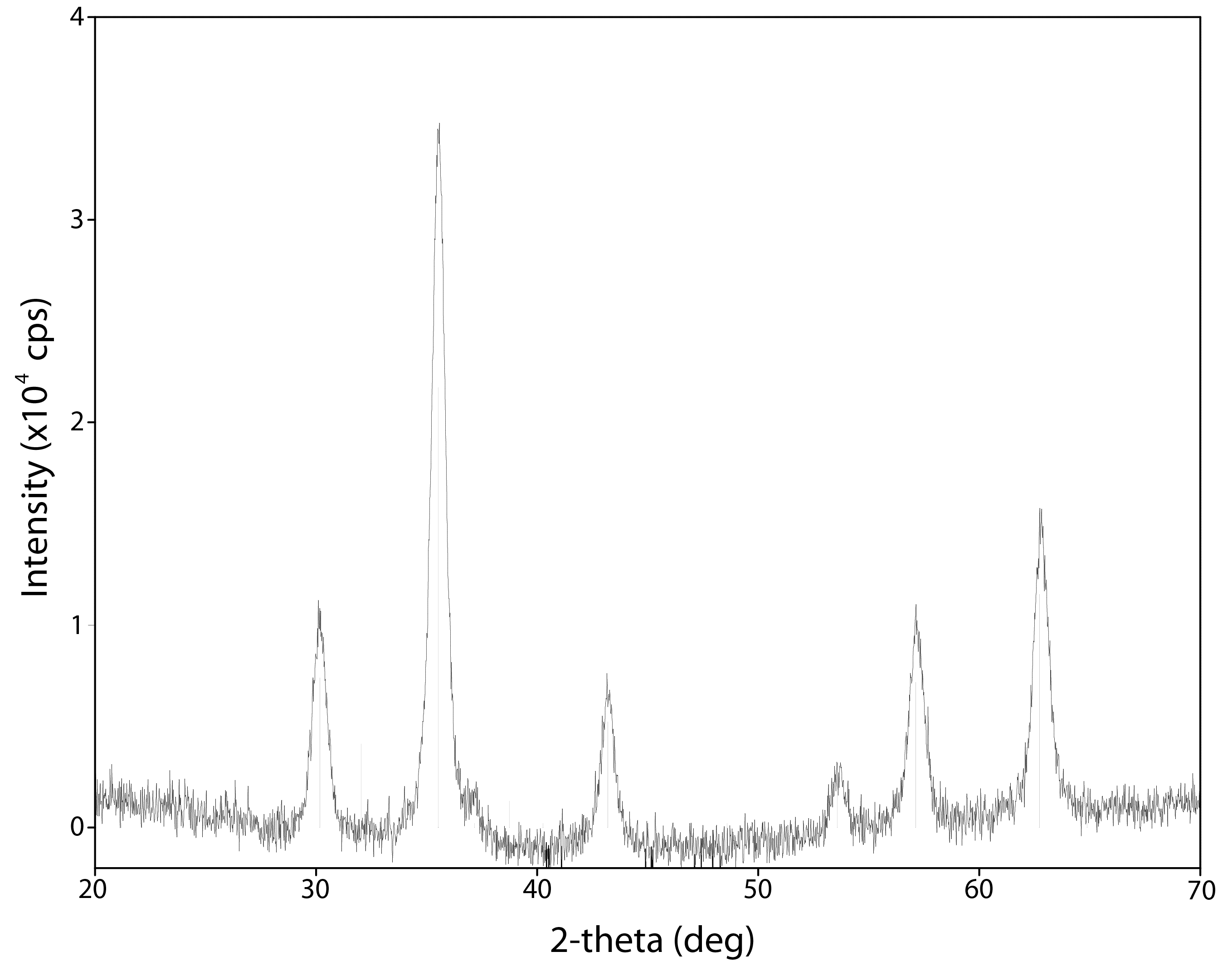


Figure S11 X-ray diffractograms (XRD) of SPIONs. Diffraction pattern of SPIONs has six characteristic diffraction peaks with indices (220), (311), (400), (422), (511), and (440), corresponding to pure iron oxide maghemite and/or magnetite.
